# Prediction of species composition ratios in pooled specimens of the *Anopheles* Hyrcanus group using quantitative sequencing

**DOI:** 10.1186/s12936-021-03868-y

**Published:** 2021-08-06

**Authors:** Do Eun Lee, Heung-Chul Kim, Sung-Tae Chong, Terry A. Klein, Ju Hyeon Kim, Si Hyeock Lee

**Affiliations:** 1 Department of Agricultural Biotechnology, Seoul National University, Seoul, 08826 Republic of Korea; 2 Medical Department Activity-Korea/65th Medical Brigade, Force Health Protection and Preventive Medicine, Unit 15281, APO, 96271-5281 AP USA; 3 Research Institute of Agriculture and Life Sciences, Seoul National University, Seoul, 08826 Republic of Korea

**Keywords:** *Anopheles* Hyrcanus Group, Species composition, *COI*, *ITS2*, Quantitative sequencing

## Abstract

**Background:**

*Plasmodium vivax* is transmitted by members of the *Anopheles* Hyrcanus Group that includes six species in the Republic of Korea: *Anopheles sinensis sensu stricto* (*s.s*.), *Anopheles pullus*, *Anopheles kleini*, *Anopheles belenrae*, *Anopheles lesteri*, and *Anopheles sineroides*. Individual *Anopheles* species within the Hyrcanus Group demonstrate differences in their geographical distributions, vector competence and insecticide resistance, making it crucial for accurate species identification. Conventional species identification conducted using individual genotyping (or barcoding) based on species-specific molecular markers requires extensive time commitment and financial resources.

**Results:**

A population-based quantitative sequencing (QS) protocol developed in this study provided a rapid estimate of species composition ratios among pooled mosquitoes as a cost-effective alternative to individual genotyping. This can be accomplished by using species- or group-specific nucleotide sequences of the *mitochondrial cytochrome C oxidase subunit I* (*COI*) and the *ribosomal RNA internal transcribed spacer 2* (*ITS2*) region as species identification alleles in a two-step prediction protocol. Standard genomic DNA fragments of *COI* and *ITS2* genes were amplified from each *Anopheles* species using group-specific universal primer sets. Following sequencing of the *COI* or *ITS2* amplicons generated from sets of standard DNA mixtures, equations were generated via linear regression to predict species-specific nucleotide sequence frequencies at different positions. Species composition ratios between *An. sineroides*, *An. pullus* and *An. lesteri* were estimated from QS of the *COI* amplicons based on the *mC*.260A, *mC*.122C and *mC*.525C alleles at the first step, followed by the prediction of species composition ratios between *An. sinensis*, *An. kleini* and *An. belenrae* based on QS of the *ITS2* amplicons using the *rI*.370G and *rI*.389T alleles. The *COI* copy number was not significantly different between species, suggesting the reliability of *COI*-based prediction. In contrast, *ITS2* showed a slightly but significantly higher copy number in *An. belenrae*, requiring an adjustment of its predicted composition ratio. A blind test proved that predicted species composition ratios either from pooled DNA specimens or pooled mosquito specimens were not statistically different from the actual values, demonstrating that the QS-based prediction is accurate and reliable.

**Conclusions:**

This two-step prediction protocol will facilitate rapid estimation of the species composition ratios in field-collected *Anopheles* Hyrcanus Group populations and is particularly useful for studying the vector ecology of *Anopheles* population and epidemiology of malaria.

**Supplementary Information:**

The online version contains supplementary material available at 10.1186/s12936-021-03868-y.

## Background

The *Anopheles* Hyrcanus Group in the Republic of Korea (ROK) includes five species (*Anopheles sinensis*, *Anopheles pullus*, *Anopheles kleini*, *Anopheles belenrae*, and *Anopheles lesteri*), which cannot be identified morphologically, and another member, *Anopheles sineroides*, which can be identified morphologically when specimens are not damaged during the collection process [1, 2]. Species identification is important because individual species within the Hyrcanus Group overlap geographically and demonstrate differences in their seasonal distributions, vector competence, and insecticide resistance [3–5]. Based on preliminary studies, *An. kleini*, and *An. lesteri* are primary vectors of *Plasmodium vivax* in the ROK, whereas *An. pullus, An. belenrae*, and *An. sinensis* are poor vectors [3–5]. As *An. kleini* is more commonly collected near the demilitarized zone (DMZ) where the majority of malaria cases occur, the density of this vector species is a primary covariate for identifying vivax malaria risk [6]. Since the L1014F mutation of voltage-sensitive sodium channel associated with pyrethroid insecticide resistance has been found only in *An. sinensis*, the efficacy of vector control may depend on the relative composition of vector species [7]. Therefore, information on the abundance of Hyrcanus Group member species in areas of likely malaria transmission is crucial for understanding the population dynamics of vector populations and epidemiology of malaria for the development and implementation of an efficient vector management strategies.

Since five members of the Hyrcanus Group cannot be identified morphologically, DNA barcoding based on the *internal transcribed spacer 2* (*ITS2*) markers have been widely used for species identification [8, 9]. However, this molecular identification is based on individual genotyping requiring extensive labour and financial resources to conduct individual specimen DNA extraction, PCR, and sequencing gene fragments for large numbers of mosquitoes. Recently, detection of *Plasmodium* species and insecticide resistance genes have been routinely conducted using pooled mosquito specimens, where > 30 mosquitoes are homogenized, DNA extracted, and processed for subsequent analyses [10, 11]. If the species-specific molecular loci are identified and a protocol to distinguish and quantify their frequencies is developed, the DNA or specimen pooling technique can be employed to estimate the proportion of each species within a species complex.

Species-specific loci of the *mitochondrial cytochrome C oxidase subunit I* (*COI*) gene and the* ITS2 rRNA* gene that can distinguish each member of the Hyrcanus Group present in the ROK were first identified. Subsequently, a quantitative sequencing (QS) protocol was developed to estimate the proportion of each species in pooled samples using individual species-specific nucleotide signals at multiple loci of the *COI* or *ITS2* genes. This two-step method was shown to provide a rapid and reliable estimation of the species composition ratios for members of the Hyrcanus Group, and thus is useful for studying vector ecology and epidemiology of vivax malaria in the ROK.

## Methods

### ***Anopheles*** genomic DNA extraction and target gene amplification

Six members of the Hyrcanus Group were collected from Paju, Gyeonggi province, ROK. For molecular identification, genomic DNA (gDNA) was individually extracted using DNeasy Blood & Tissue Kit (QIAGEN, Germany). Then, the *ITS2* region of each specimen was amplified using rDNA 5.8 S forward (5′-TGTGAACTGCAGGACACATGAA-3′) and rDNA 28 S reverse (5′-ATGCTTAAATTTAGGGGGTAGTC-3′) primers [12]. The reaction mixture (25 µl) contained 10 ng of template DNA, 2 µl of 2.5 mM dNTP, 2.5 µl of 10X buffer, 0.4 µM of each primer, 0.12 µl of EX Taq polymerase (Takara Biotechnology, Japan) and double distilled water (ddH_2_O). A 3 min preincubation at 95 ℃ was followed by 34 cycles at 95 ℃ for 20 s, 55 ℃ for 30 s, and 72 ℃ for 1 min, with a final extension at 72 ℃ for 5 min. PCR products were purified using a Monarch Clean up kit (New England Biolabs, USA) and sequenced using an ABI3730xl sequencer at the National Instrumentation Center for Environmental Management (NICEM, Korea). Sequences from each *Anopheles* specimen were submitted as queries to Basic Local Alignment Search Tool (BLAST) to search similar data in GenBank. A maximum-likelihood (ML) phylogenetic tree for the *ITS2* sequences (455–492 bp) for each of the six Hyrcanus Group species was created along with the reference sequences (1624–1651 bp) obtained from GenBank using MEGA-X (ver.10.0.5) (iGEM, USA).

### *COI* and *ITS2* sequence alignment

Although *COI* gene is a generally used marker for the identification of mosquito species, *ITS2* was additionally used since the differences in COI sequence were insufficient to distinguish all the sibling species within Hyrcanus group. To detect any intra-species sequence polymorphism, five to seven COI sequences and three to five *ITS2* sequences of each Hyrcanus Group species were downloaded from National Center for Biotechnology Information (NCBI) (Additional file 1). *COI* sequences and *ITS2* sequences were aligned respectively using DNAstar MegAlign software (DNASTAR Inc., USA) by ClustalW methods. The *ITS2* sequences obtained from collected mosquito samples were also aligned with downloaded sequences. From the alignment data, species-specific or group-specific nucleotide sequences were identified (Table 1).

**Table. 1:** Species-specific nucleotide sequence loci of *Anopheles* species in *COI* and *ITS2*

*COI*		*mC*.122	*mC*.260	*mC*.387	*mC*.443	*mC*.525	*mC*.527	*mC*.582	*mC*.590
*An. pullus*		**C**	T	**C**	A	T	A	**C**	T
*An. lesteri*		T	T	T	A	T	A	T	T
*An. sineroides*		**C**	**A**	T	**T**	T	A	T	T
*skb*^a^		T	T	T	A	**C**	**T**	T	**C**

*ITS2*		*rI*.370	*rI*.372	*rI*.377	*rI*.378	*rI*.380	*rI*.384	*rI*.389	*rI*.400
*An. sinensis*		A	C	**T**	A	C	T	A	G
*An. kleini*		**G**	**T**	C	A	**T**	**G**	A	G
*An. belenrae*		A	C	C	**G**	C	T	**T**	**A**

Species-specific nucleotides were screened from *CO1* and* ITS2* alignment for members of the *Anopheles* Hyrcanus Group

^a^ *skb* = *An. sinensis, An. kleini* and *An*. *belenrae*

### QS primer design for the amplification of *COI* and *ITS2* fragments

A set of primers (An_*COI*-F and An_*COI*-R) were designed from the conserved sequence regions across all six *Anopheles* species to equally amplify the target *COI* fragments among each of the mosquito species (Table 2). For *ITS2* amplification, a set of primers (*Anskb_ITS2*-F and *Anskb_ITS2*-R) were designed from the conserved sequence regions of *An. sinensis*, *An. kleini*, and *An. belenrae* to block the amplification in *An. sineroides*, *An. pullus*, and *An. lesteri* (36 ~ 45 % sequence identity for *Anskb*_*ITS2*-F; 68.2 % sequence identity for *Anskb*_*ITS2*-R).

**Table. 2:** Designed primer sets used for predicting relative species composition ratios

Gene	Primer name	Sequence (5′–3′)	Size (bp)
*COI*	*An_COI*-F	CTTTAAGTATTCTAATTCGAGCTG	594
	*An_COI*-R	TAAAATWGGRTCTCCTCCTCC	
*ITS2*	*Anskb_ITS2*-F	CAGACAAGTAGAAAGGGCTGT	234^a^/235^b^/238^c^
	*Anskb_ITS2*-R	ACAAATCTGGGTAGTGTTCTCT	

^a^*Ank* = *An. kleini*

^b^*Anb* = *An. belenrae*

^c^*Ans *= *An. sinensis*

The target DNA fragments were amplified from pooled DNA samples using the *An_COI*-F vs. *An_COI*-R and *Anskb_ITS2*-F vs. *Anskb_ITS2*-R primer sets, respectively. The *COI* amplification reaction mixture contained 20 ng of each gDNA template, 0.2 mM of dNTP, 2.5 µl of 10X buffer, 0.5 µM of each primer, 0.12 µl of EX Taq polymerase (Takara), and ddH_2_O up to 25 µl. PCR cycling conditions included preincubation for at 95℃ for 3 min, followed by 32 cycles at 95℃ for 20 s, 56℃ for 30 s, and 72℃ for 1 min, with a final extension at 72℃ for 5 min. To amplify the* ITS2* fragment from three species (*An. sineroides*, *An. pullus*, and *An. lesteri*), the reaction mixture contained 10 ng of gDNA template, 0.2 mM of dNTP, 2.5 µl of 10X buffer, 0.25 µM of each primer, 4 % of DMSO, 0.12 µl of EX Taq polymerase, and ddH_2_O up to 25 µl. PCR cycling conditions included a preincubation at 95℃ for 3 min, followed by 34 cycles at 95 ℃ for 20 s, 64℃ for 25 s, 72 ℃ for 50 s, with a final extension at 72 ℃ for 5 min.

### Establishment of a two-step QS protocol for estimating species composition ratios

**Fig. 1:**
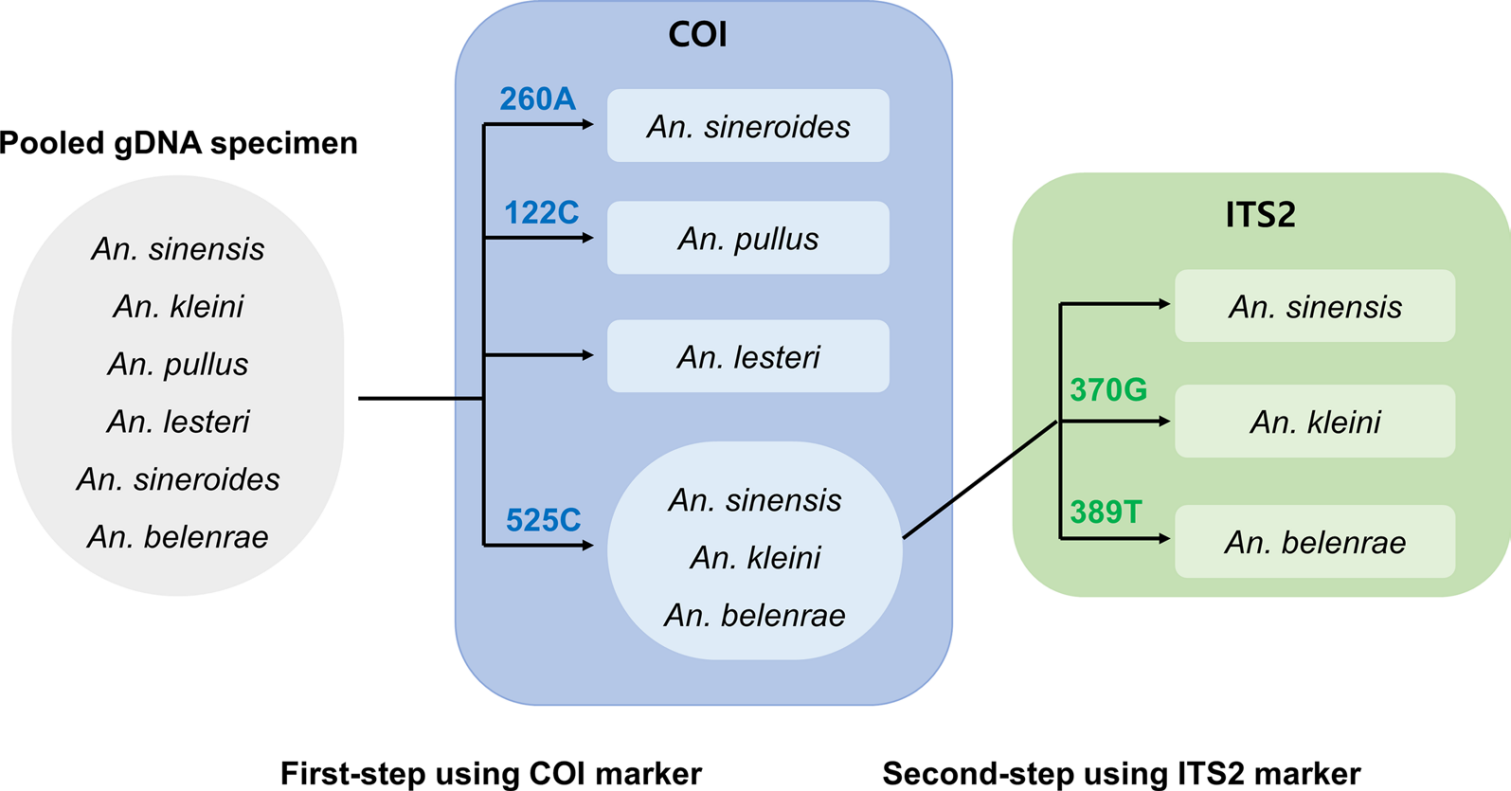
Schematic diagram of the workflow for predicting species composition ratio. The *COI* fragment were amplified and sequenced from pooled gDNA specimens. Based on the sequencing chromatograms, the nucleotide signals of three *COI* loci were quantified to estimate the composition ratios of *Anopheles sineroides*, *Anopheles pullus*, and *Anopheles lesteri*. Next, the *ITS2* gene fragments were selectively amplified and sequenced for *Anopheles sinensis*, *Anopheles kleini*, and *Anopheles belenrae*. The nucleotide signals of two *ITS2* loci were quantified from the sequence chromatograms and the composition ratios of *An. sinensis*, *An. kleini*, and *An. belenrae* were deduced

A two-step QS workflow was developed using PCR-amplified fragments of the *COI* and *ITS2* genes (Fig. 1). The species composition ratios between *An. sineroides*, *An. pullus*, and *An. lesteri* were first estimated using QS of the *COI* amplicons that included target DNA fragments from all the six *Anopheles* species, if present. The species composition ratios of *An. sinensis*, *An. kleini*, and *An. belenrae* were predicted using QS of the *ITS2* amplicons that did not contain amplified target DNA fragments from *An. sineroides*, *An. pullus*, or *An. lesteri*.

Based on the characteristics of Sanger sequencing that the nucleotide signal intensity is affected by the surrounded nucleotide bases [13], equally diluted PCR products of each species were mixed in various ratios to prepare standard DNA templates for QS (Additional file 2) and sequenced to establish the nucleotide signal prediction equations based on linear regression analysis. In preparing the standard DNA templates, the proportion of *An. sineroides* was limited to 10–50 % since collected numbers are usually < 5 % of all *Anopheles* species collected in the ROK [14], whereas other species were mixed at 10–90 % proportions. Nucleotide base signal ratios in sequencing data were analysed using Chromas (ver. 2.6.6) and linear regression analysis was done using GraphPad Prism (ver. 6, GraphPad Inc., USA). Linear regression analysis was performed for all the species-specific nucleotide loci of *COI* and *ITS2* to select the most reliable locus based on the R^2^ criteria and standard error of estimate (*S*_*est*_), which were the statistical measures for goodness-of-fit.

### Determination of copy numbers of *COI* and *ITS2*

Copy numbers of *COI* (or mitochondria number) and *ITS2* loci were measured by quantitative real-time PCR (qPCR) using the LightCycler 96 (Roche, Basel, Switzerland). *Ribosomal protein S7* (*RPS7*) and *ribosomal protein L8* (*RPL8*) were used as reference genes since they are known as single-copy genes [15, 16]. gDNA was extracted from individual mosquitoes, and species was identified based on *ITS2* markers [8] as described earlier. Following species identification, 3 ~ 5 individual gDNA samples were combined and used as templates for qPCR. The qPCR reaction mixture (10 µl) contained 10 ng of gDNA template, 0.5 µM of each primer (Additional file 3) and 5 µl of TB Green™ Premix Ex Taq™ II (Takara biotechnology, Japan). qPCR for each species was conducted with 7 ~ 20 replicates using the amplification condition of 95 °C denaturation step for 1 min followed by 35 cycles of 95 °C for 15 s, 57 °C for 20 s, and 72 °C for 30 s. Melting curve analysis was conducted to confirm the integrity of PCR product by starting the melting step at 60 °C and increasing to 95 °C with a ramping temperature of 0.2 °C/s. The copy number of *COI* was estimated for all the six mosquito species. In case of *ITS2*, however, the copy number was only determined for *An. sinensis*, *An. kleini* and *An. belenrae*, as *ITS2* was used for the prediction of species composition ratio in the three species.

### Verification assay using pooled DNA and pooled mosquito specimens

Based on the previous study that the primary *Anopheles* species collected in various traps in the ROK are *An. sinensis*, *An. kleini*, and *An. pullus*, the accuracy of the QS protocol was evaluated with serial gDNA mixtures of the three species. The gDNA ratios between two species in all the three combinations (*An. pullus*:*An. sinensis*, *An. pullus*:*An. kleini*, and *An. sinensis*:*An. kleini*) were prepared at ratios of 2:8, 3:7, 4:6, 6:4, 7:3 and 8:2, and three species combinations of 2:3:5 and 1:2:7 was amplified using *COI* and *ITS2* primer sets. The signal intensity was obtained from two nucleotide positions of *COI* [*mC*.122 (the nucleotide number 122 of *mitochondrial COI* gene; the same rule of nomenclature applies hereafter) and *mC*.525] and a single locus (*rI*.370; the nucleotide number of 370 for ribosomal genes in the* ITS2* region; the same rule of nomenclature applies hereafter) of* ITS2*. Predicted composition ratios were obtained by inversely substituting the gDNA ratios into regression equations (Tables 3, 4). The observed values of the three species were calculated by the peak of the signal intensity chromatogram. The Pearson correlation coefficient (*r*) and errors between observed and predicted values were calculated using GraphPad Prism (Table 5).

To confirm that the established protocol is valid with gDNA samples prepared from pooled mosquito specimens, groups of 20 ~ 24 individual mosquitoes collected from six different collection times and two different locations (DMZ-0619, 0712, 0828, 0911; Pyeongtaek-0706, 0815) were randomly selected and pooled. gDNA was released from one wing of each specimen using DNA releasing buffer (5 % of DMSO, 5 % of PEG 200, 20 mM of NaOH, and 1 mM of EDTA) for species identification. In brief, each wing was incubated in 15 µl of DNA releasing buffer for 20 min at room temperature and then diluted with 25 µl of water for PCR reaction. Following species identification based on the *ITS2* marker [8], the remaining mosquito bodies were combined and homogenized together by plastic pestle in liquid nitrogen. Species composition ratios were predicted by inversely substituting the gDNA ratios into regression equations and compared to actual species composition data.

**Table. 3:** Linear regression analysis^a^ results of species-specific nucleotide position of *COI* gene

Locus	Species distinction	Nucleotide	N^b^	R^2^	*S*_*est*_^c^	y = f(x)
*mC*.260A	*An. sineroides*	A	6	0.995	1.49	1.039x + 1.446
*mC*.443T	*An. sineroides*	T	6	0.993	1.71	0.9831x + 1.910
*mC*.122C	*An. sineroidesAn. pullus*	C	14	0.997	1.78	0.9942x + 2.369
*mC*.387C	*An. pullus*	C	11	0.995	2.49	1.051x + 0.289
*mC*.582C	*An. pullus*	C	11	0.985	4.26	1.027x + 1.412
*mC*.525C	*skb*	C	11	0.998	1.51	1.008x + 0.0612
*mC*.527T	*skb*	T	11	0.998	1.63	0.9903x – 1.457
*mC*.590C	*skb*	C	11	0.991	3.24	0.9821x + 2.635

^a^Linear regression analyses for 8 nucleotide positions were done by GraphPad Prism. The selected markers and corresponding equations for species distinction are underlined

^b^The number of x values used for regression analysis

^c^Standard error of estimate

**Table. 4:** Linear regression analysis^a^ results of species-specific nucleotide position of *ITS2* gene

Locus	Species distinction	Nucleotide	N^b^	R^2^	*S*_*est*_^c^	f(x)
*rI*.370G	*An.kleini*	G	9	1.000	0.65	0.998x – 0.547
*rI*.372T	*An.kleini*	T	9	0.993	3.29	1.029x − 5.815
*rI*.380T	*An.kleini*	T	9	0.968	6.83	1.012x + 7.43
*rI*.377T	*An.sinensis*	T	9	0.894	12.4	0.933x + 10.0
*rI*.378G	*An.belenrae*	G	9	0.996	2.28	1.003x + 0.071
rI.389T	*An.belenrae*	T	9	0.999	1.33	0.996x + 1.038
*rI*.400A	*An.belenrae*	A	9	0.865	14.0	1.042x -3.667

^a^Linear regression analyses for 8 nucleotide positions were done by GraphPad Prism. The selected markers and corresponding equations for species distinction are underlined

^b^The number of x values used for regression analysis

^c^Standard error of estimate

**Table. 5:** Evaluation of the accuracy of the predicted composition ratios from the pooled gDNA and pooled mosquito specimens

Specimen condition	Data set	Number of data points	*r*^a^	*p*	R^2^	MAE^b^	Min-max error
Pooled gDNA specimen	All	45	0.984	< 0.0001	0.968	3.87	0.10–12.2
	*COI*	21	0.977	< 0.0001	0.955	4.45	0.10–12.2
	*ITS2*	30	0.982	< 0.0001	0.965	4.02	0.12–12.2
	*An. pullus*	15	0.992	< 0.0001	0.984	3.57	0.10–7.34
	*An. kleini*	15	0.991	< 0.0001	0.982	3.28	0.15–10.8
	*An. sinensis*	15	0.983	< 0.0001	0.966	4.76	0.12–12.2
Pooled mosquito specimen	All	36	0.985	< 0.0001	0.971	2.90	0.06–13.2
	*COI*	24	0.987	< 0.0001	0.974	3.59	0.19–13.2
	*ITS2*	18	0.984	< 0.0001	0.968	2.21	0.06–12.8
	*An. pullus*	6	0.985	0.0002	0.970	4.36	0.17–10.9
	*An. kleini*	6	0.985	0.0002	0.971	2.83	0.06–12.8
	*An. sinensis*	6	0.990	< 0.0001	0.980	3.15	0.16–12.1
	*An. belenrae*	6	0.997	< 0.0001	0.994	0.66	0.07–1.56
	*An. lesteri*^*c*^	6	N/A	N/A	N/A	3.85	1.51–8.13
	*An. sineroides*	6	0.984	0.0002	0.968	1.09	0.19–1.45

^a^Pearson correlation coefficient

^b^Mean absolute error

^c^Statistical analysis of *An. lesteri* was not possible since all the actual value was zero

## Results

### Phylogenetic tree of collected specimens

Based on the *COI* phylogenetic tree, *An. sineroides*, *An. pullus*, and *An. lesteri* were clearly divided into separate clusters, whereas *An. sinensis*, *An. kleini*, and *An. belenrae* were clustered into a large monophyletic cluster (Additional file 4). In contrast, the *ITS2* phylogenetic analysis demonstrated that all the collected specimens were clearly divided into separate clusters with corresponding GenBank references (Additional file 5).

### Search for species-specific loci in *COI* and *ITS2*

The results of *COI* and *ITS2* sequence alignment were organized with different color codes for each species (Additional files 6, 7). Residues that match the consensus sequences were marked as dots, and black background was applied for the sequences that differ from the consensus. The locations of species-specific *COI* sequence loci used for species discrimination are listed in Table 1. Nucleotide sequences at seven *COI* loci were found to be either species- or group-specific. The *mC*.122C (cytosine at the nucleotide number 122 of *mitochondrial COI* DNA) was only observed in *An. pullus*, and *An. sineroides*, whereas the remaining four species had *mC*.122T. At the *mC*.260, and *mC*.443 loci, *An. sineroides* was separated from other species by having adenine and cytosine, respectively, thus allowing *mC*.260A (adenine at the *mC*.260 locus; the same rule of nomenclature applies hereafter) and *mC*.525C as *An. sineroides*-specific alleles. The cytosine nucleotide bases at both *mC*.387 and *mC*.582 nucleotide positions were only specific to *An. pullus*, thus these alleles were used as *An. pullus*-specific markers. No species-specific nucleotides to *An. sinensis*, *An. kleini, An. belenrae*, or *An. lesteri* were found at any *COI* loci examined. However, group-specific nucleotides (*mC*.525C, *mC*.527T, and *mC*.590C) were identical in all three species of *An. sinensis*, *An. kleini*, and *An. belenrae*.

Nucleotide sequence alignment of ribosomal RNA genes from *An. sinensis*, *An. kleini*, and *An. belenrae* demonstrated that the longest fragment without any insertion/deletion (indel) was located in the region containing *5.8S rDNA* and *28S rDNA* of the *ITS2* region (nucleotide number 208–446 of *An. sinensis*). Among a total of eight *ITS2* nucleotide positions specific to individual species, the *rI*.377T (thymine at the nucleotide number 377 of ribosomal DNA *ITS2* region; the same rule of nomenclature applies hereafter) was only specific to *An. sinensis*, whereas other two species had *rI*.377 C (Table 1). The nucleotide sequences at four positions (*rI*.370G, *rI*.372T, *rI*.380, and *rI*.384) were specific to *An. kleini*, whereas those at three nucleotide positions (*rI*.378G, *rI*.389T, and *rI*.400A) were specific to *An. belenrae* (Table 1).

**Fig. 2:**
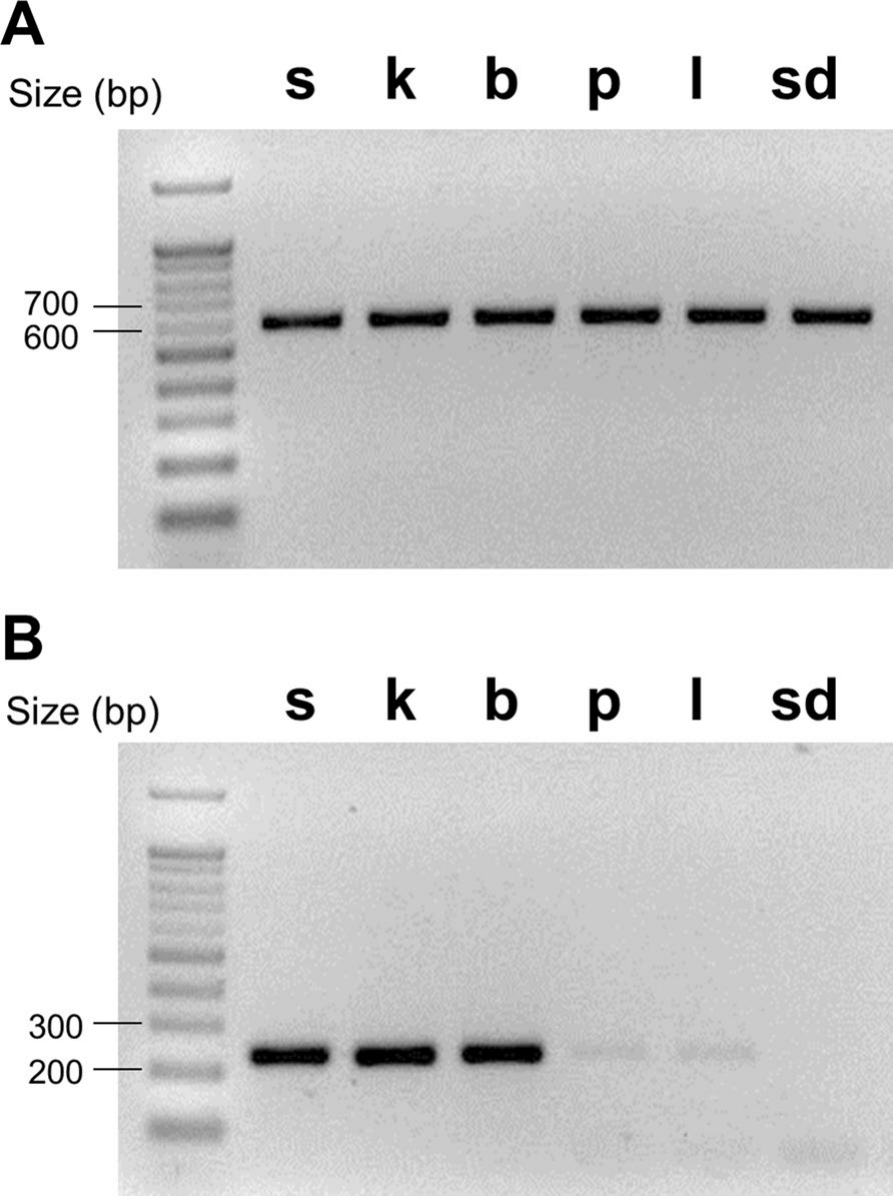
Amplification of gDNA fragments of *COI* and *ITS2* genes. **A** The gDNA fragments of *COI* gene (594 bp) were equally amplified from all the six *Anopheles* species (s = *An. sinensis*; k = *An. kleini*; b = *An. belenrae*; p = *An. pullus*; l = *An. lesteri*; sd = *An. sineroides*). **B** The gDNA fragments of* ITS2* gene (234–238 bp) were amplified from *An. sinensis*, *An. kleini*, and *An. belenrae*, but not amplified from *An. pullus*, *An. lesteri*, and *An. sineroides*

The target *COI* fragment was equally amplified from all six species (Fig. 2A). Due to the substantial differences in the priming sequences when using the *Anskb_ITS2*-F and R primers, however, the *ITS2* fragment was only amplified from *An. sinensis*, *An. kleini*, and *An. belenrae* but not from *An. sineroides*, *An. pullus*, and *An. lesteri* (Fig. 2B).

### Establishment of prediction equation

**Fig. 3:**
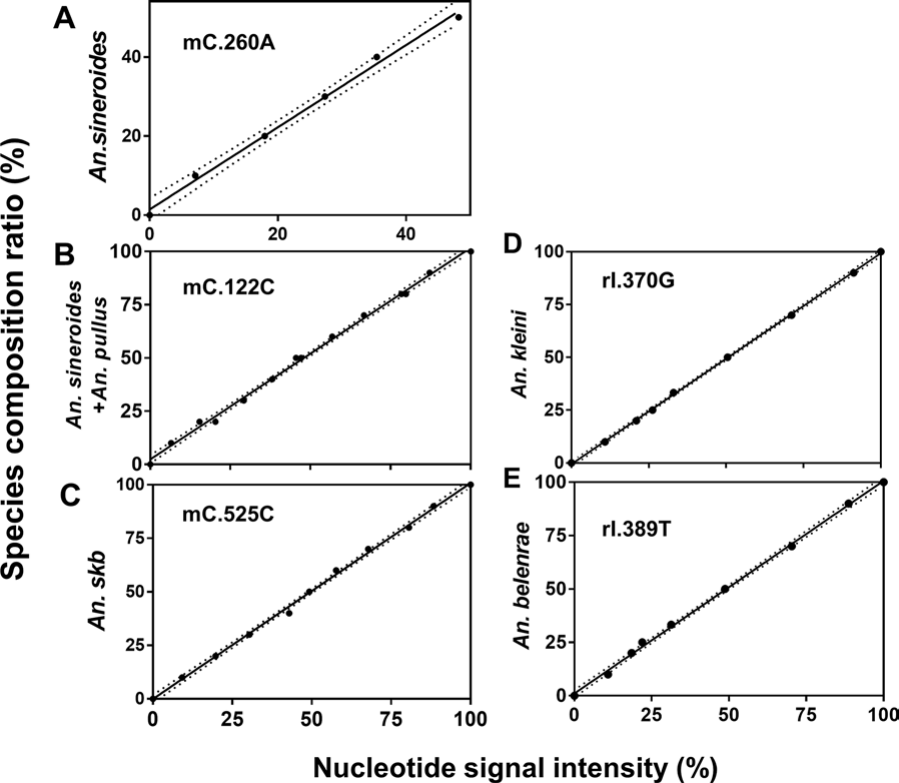
Linear regression equations for the prediction of species-specific nucleotide frequencies at five different loci of *COI* and *ITS2*. Relationships between nucleotide signal intensities and species compositions of (**A**) *An. sineroides*, (**B**) *An. sineroide*s + *An. pullus*, (**C**) *An. sinensis + An. kleini + An. belenrae*, (**D**) *An. kleini*, and (**E**) *An. belenrae* with 95 % CI in dotted lines

Linear regression analysis was performed for all the eight species-specific nucleotide loci of *COI* to select the most reliable loci based on the criteria (R^2^ and *S*_*est*_) (Table 3). Between the two loci specific to *An. sineroides* (*mC*.260 and *mC*.443), the *mC*.260 locus was selected as the species-specific locus for the identification of *An. sineroides* because it was determined to be more reliable by showing better criteria values (0.995 and 1.49 for R^2^ and *S*_*est*_, respectively). In the case of two alleles specific to *An. pullus* (*mC*.387C and *mC*.582C), they were excluded as species distinction alleles, since their *S*_*est*_ values (2.49 at *mC*.387C and 4.26 at *mC*.582C) were much larger than those of other loci. Instead, the *mC*.122C, showing better criteria values (0.997 and 1.78 for R^2^ and *S*_*est*_, respectively), was selected as an alleles that can simultaneously distinguish both *An. pullus* and *An. sineroides* from other species. Among the three alleles specific to the combined group of *An. sinensis*, *An. kleini*, and *An. belenrae* (*mC*.525C, *mC*.527T, and *mC*.590C), *mC*.525C was determined to be the best allele to estimate the combined proportion of *An. sinensis*, *An. kleini*, and *An. belenrae* out of six candidate species (Fig. 3A–C). Since no nucleotide position was found to be only specific to *An. lesteri*, the composition of *An. lesteri* was deduced by subtracting the combined ratios of the other five species from 1.

The same analysis and screening were performed for the seven candidate nucleotide positions (*rI*.384 was excluded due to the unstable signal intensity) in the *ITS2* amplicon (Table 4). Since the *rI*.370 nucleotide position exhibited the best criteria scores (1.00 and 0.65 for R^2^ and *S*_*est*_, respectively) for *An. kleini* distinction among the three nucleotide positions (*rI*.370, *rI*.372 and *rI*.380), the* rI*.370G was used as the allele to estimate the ratio of *An. kleini*. Likewise, the *rI*.389T allele (0.999 and 1.33 for R^2^ and *S*_*est*_, respectively) was selected out of the three alleles (*rI*.378G, *rI*.389T, and *rI*.400A) for estimating the proportion of *An. belenrae* (Fig. 3D, E). Because the *An. sinensis*-specific *rI*.377T position produced relatively lower criteria scores (0.894 and 12.4 for R^2^ and *S*_*est*_, respectively), it was not used to estimate the proportion of *An. sinensis*. Instead, the proportion of *An. sinensis* was calculated by subtracting the combined proportions of *An. kleini* and *An. belenrae* from the total proportions of *An. sinensis*, *An. kleini*, and *An. belenrae*.

### Copy numbers of *COI *and *ITS2* loci between species in the ***Anopheles*** Hyrcanus group

**Fig. 4:**
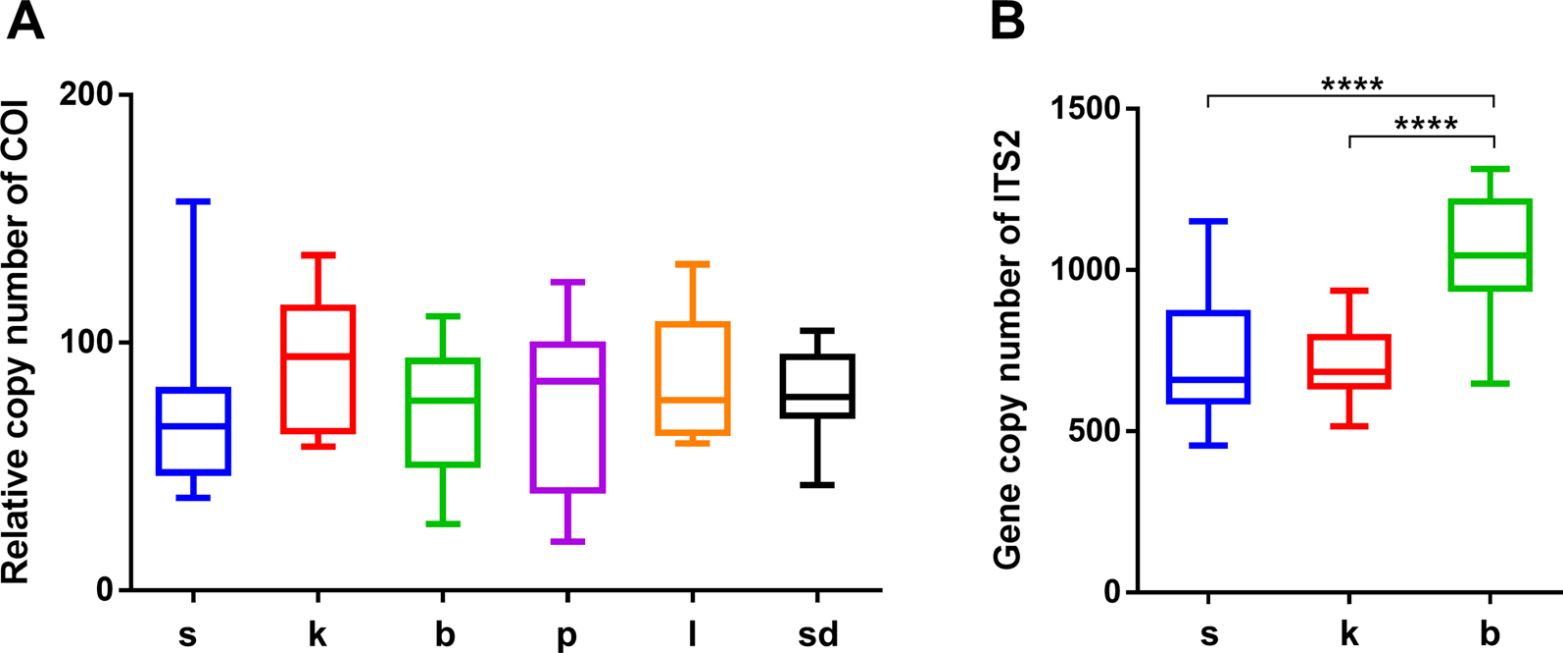
Copy numbers of *COI* and *ITS2* loci of *Anopheles* Hyrcanus Group species. **A** The relative copy numbers of *COI* locus of six *Anopheles* species (s = *An. sinensis*; k = *An. kleini*; b = *An. belenrae*; p = *An. pullus*; l = *An. lesteri*; sd = *An. sineroides*). **B** The relative copy number of *ITS2* locus of *An. sinensis*, *An. kleini*, and *An. belenrae*

The copy numbers of *COI* gene, which reflect the relative numbers of mitochondria, ranged from 69.9 (*An. lesteri*) to 99.8 (*An. kleini*), but were not significantly different between all the six *Anopheles* species examined (*p* = 0.232; Fig. 4A). However, the average *ITS2* copy number of *An. sinensis*, *An. kleini* and *An. belenrae* was 795, 689 and 1054, respectively. The *ITS2* copy number of *An. belenrae* was significantly higher (1.36-fold, *p* < 0.0001) from the other two species (Fig. 4B). Since the nucleotide signal ratio of *rI*.377T site can be overestimated by the copy number factor of 1.36, the predicted value of *An. belenrae* composition ratio was further adjusted by multiplying the factor of 0.7.

### Evaluation of the QS accuracy

**Fig. 5:**
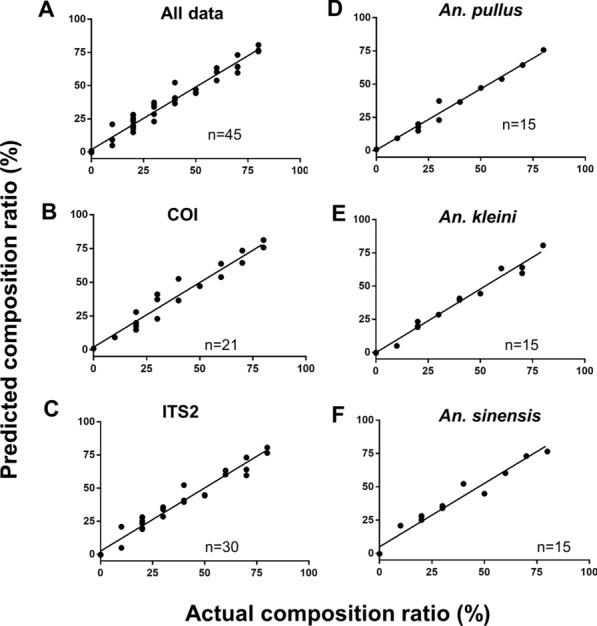
Evaluation of accuracy between the actual and predicted composition ratios from pooled gDNA specimens. The x axis indicates actual species composition ratios obtained from gDNA proportions, whereas the y axis was predicted species composition ratios deduced from the prediction equations. A total of 45 estimates were obtained from the verification test and plotted against their corresponding actual values. The graphs show the linear regression between the actual and predicted values with (**A**) all data points, (**B**) values from *COI* gene, (**C**) values from* ITS2* gene, and (**D** ~** F**) values from *An. pullus*, *An. kleini*, and *An. sinensis*

Validation of the QS method of three major species were performed using 15 sets of gDNA mixtures. To verify the correlation and error between the actual and predicted values, a total of 45 sets of data were plotted on a graph with an x-axis representing the actual species composition ratio and a y-axis representing the estimated ratios (Fig. 5A). In addition, separate analyses were conducted for the 21 and 30 data sets derived from *COI* and *ITS2* genes, respectively, and 15 data sets from the major species composed of three members of the Hyrcarus Group (*An. pullus*, *An. kleini*, and *An. sinensis*) (Fig. 5B–F). Overall data sets were confirmed to have significant correlations (*r* > 0.977, *p* < 0.001, R^2^ > 0.955) between the actual and predicted composition ratios (Table 5). Mean absolute errors (MAE) of the QS prediction data of the three major species was 3.87 %, and MAE of *COI *and *ITS2* loci were 4.45 and 4.02 %, respectively. The MAE rates for the prediction of *An. pullus, An. kleini*, and *An. sinensis* were 3.57 % (maximum 7.34 %), 3.28 % (maximum 10.8 %), and 4.76 % (maximum 12.2 %), respectively.

**Fig. 6:**
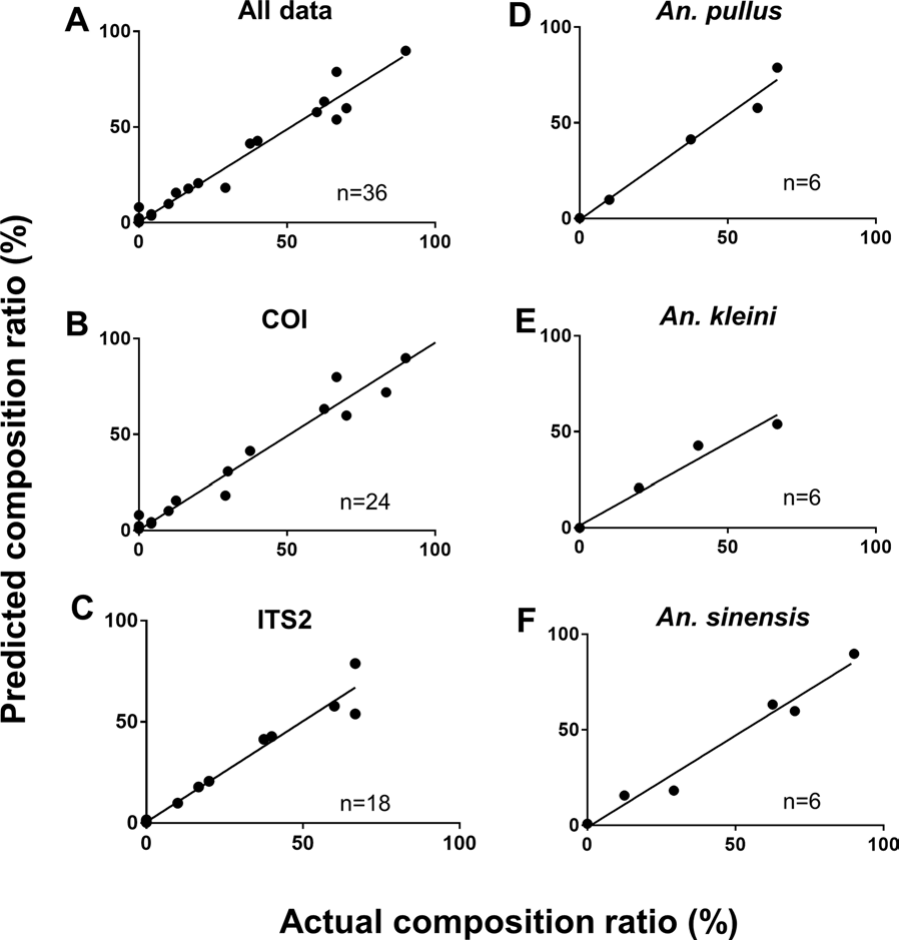
Evaluation of accuracy between the actual and predicted composition ratios from pooled mosquito specimens. The x axis indicates actual species composition ratios obtained from individually identified mosquitoes in pooled mosquito specimens, whereas the y axis was predicted species composition ratios deduced from the prediction equations. A total of 36 estimates were obtained from the verification test and plotted against their corresponding actual values. The graphs show the linear regression between the actual and predicted values with (**A**) all data points, (**B**) values from *COI* gene, (**C**) values from *ITS2* gene, and (**D**–**F**) values from *An. pullus*, *An. kleini*, and *An. sinensis*

Validation experiments were also conducted using gDNA samples prepared from pooled mosquito specimens. Five species (*An. sinensis*, *An. kleini*, *An. pullus*, *An. belenrae*, and *An. sineroides*) were found in pooled mosquito populations with composition ratios of 0:16:3:4:1, 0:9:5:6:0, 16:0:6:0:1, 1:0:9:0:0, 3:0:5:0:0 and 3:2:0:0:0, respectively, as determined by individual genotyping (Additional file 8). When the actual and predicted data sets were plotted on the graph (Fig. 6), the pooled mosquito data sets also showed significant correlations (*r* > 0.984, *p* < 0.0002, R^2^ > 0.968) between the actual and predicted composition ratios of *Anopheles* species (Table 5). MAE of the all data was 2.9 %, which is less than that of the pooled gDNA data. MAE values of *COI* and *ITS2* were also lower (3.59 and 2.21 %, respectively) than those from the pooled gDNA data. Since the species with low frequencies obtains lower error rates (*An. belenrae* 0.66 %, *An. sineroides* 1.09 %), their overall MAEs were lower in the pooled mosquito data compared to the pooled gDNA specimen data. The maximum prediction error generated from the all data set was estimated to 13.2 %.

## Discussion

*Anopheles* species have been conventionally identified by morphological characteristics. However, when this method fails (e.g., for members of the Hyrcanus Group in the ROK), individual genotyping has been used to identify each species, from which the relative overall species composition was determined. As a cost-effective alternative to individual genotyping (or barcoding), a population-based QS protocol was developed that can rapidly process large numbers of mosquitoes to estimate their relative species abundance. Since similar prediction error rates were found between the pooled DNA specimens and pooled mosquito specimens, mosquito samples can be pooled and processed for QS-based prediction of species composition, as previously reported in the QS-based prediction of resistance allele frequencies in head lice [17]. Since variations in DNA content and quality between species could influence the results as main error sources, however, mosquito specimens with the same body size and quality (live or preserved specimens under identical conditions) should be used for diagnosis. In addition, copy numbers of the target loci used for species prediction should be considered as they directly determine the DNA content: if one species has more copies of the DNA loci investigated, the assay would overestimate its abundance. In this study, the mitochondrial *COI* alleles could be safely employed as species distinction markers as the copy number of *COI* did not differ significantly between different species in Hyrcanus group. In case of* ITS2*, however, *An. belenrae* showed slightly higher copy numbers than *An. sinensis* and *An. kelini*, requiring an additional adjustment in the prediction of its species composition. Once wild-caught *Anopheles* are collected, 30–100 individual mosquitoes, depending on the relative size of the collections, can be pooled (whole bodies or isolated abdomens) and processed for downstream procedures, including gDNA extraction, PCR, and sequencing. Analysis of a single pooled mosquito sample provides information on the species composition that is nearly equivalent to that obtained by the multiple numbers of individual genotyping, thereby substantially saving time and resources. For example, QS-based analysis of one pool of 100 mosquitoes requires a total cost of approximately $7 and 1.5 days including sequencing cost and time, whereas separate individual genotyping with 100 individual mosquitoes would require much greater costs, particularly when sequencing is involved, and much longer time for gDNA extraction and downstream processes. This cost- and time-effectiveness is especially beneficial when processing large numbers of mosquitoes from different geographical locations and collection time points compared to individual genotyping.

Nevertheless, the information obtained from the QS-based prediction is not as accurate as that obtained from individual genotyping due to the prediction error. The prediction errors were on average 4.45 and 4.02 % when based on *COI* and *ITS2* genes, respectively. The respective composition ratio of *An. pullus*, *An. lesteri, An. kleini, An. belenrae*, and *An. sinensis* was deduced from combinations of two or three alleles (*mC*.260A vs. *mC*.122C, *mC*.122C vs. *mC*.525C, *mC*.525C vs. *rI*.370G, *mC.*525C vs. *rI*.389T and *mC.*525C vs. *rI*.370G vs. *rI.*389T, respectively). Therefore, the error for predicting the relative ratios of these species is additive and thus becomes larger than directly predicting the composition of *An. sineroides*, where species composition is deduced from a single marker. Since the maximum error rates for prediction of *An. pullus, An. kleini*, and *An. sinensis* were 10.9 %, 12.8 %, and 12.2 %, respectively, the prediction may not be accurate when the composition ratios of these species are lower than their maximum error rates. It is also worth to note that if mosquito species other than the ones investigated, such as *An. koreicus*, a rarely collected species, is present in a pool, the prediction results would be influenced. Considering the prediction error, this QS-based protocol is better suited as a primary survey tool to rapidly assess species composition in multiple pooled specimens in a tier system. If more accurate information on species composition for any particular mosquito sample is needed, a second round of analysis based on the conventional individual genotyping can be conducted [18].

Insecticide resistance for members of the Hyrcanus Group has been reported to be widely distributed in the ROK. Interestingly, as determined by QS-based genotyping, the resistance mutation frequencies fluctuated significantly throughout the mosquito season [11]. Frequencies of L1014F/C and G119S mutations associated with resistance to pyrethroid and organophosphorus insecticides, respectively, dramatically decreased in the Hyrcanus Group toward the fall and became zero the following spring, suggesting a possible overwintering cost associated with insecticide resistance. However, since the resistance mutation frequency was highly proportional to the composition of *An. sinensis* within the Hyrcanus Group ([7]; Lee DE, unpublished data), rapid estimation of the proportion of *An. sinensis* is crucial for understanding the resistance dynamics of *Anopheles* mosquitoes throughout the season. With this in mind, the high-throughput prediction of species composition based on QS using pooled DNA will facilitate the understanding of differences in insecticide resistance potential between different *Anopheles* spp.

The information on the geographical and seasonal distributions of *Anopheles* mosquitoes is crucial for establishing an efficient malaria management program. Since the species belonging to the Hyrcanus Group varies depending on geographical location and collection season, it is essential to precisely identify the relative numbers and proportion of each species over time and geographical distributions. Information on *Anopheles* species composition in northern Gyeonggi province in the ROK, considered as a high-risk area for vivax malaria, is particularly critical as *An. kleini* and *An. lesteri* were reported as the primary vectors with significantly high sporozoite rate and infection rate than *An. sinensis* [3, 19]. Distribution and predominant species change throughout the year in northern Gyeonggi province, with *An. lesteri* being predominant along the western coastal areas, whereas *An. kleini*, *An. belenrae*, and *An. sinensis* are distributed more centrally [20]. In addition, *An. pullus* and *An. belenrae* are found in early summer, *An. kleini* in mid-summer, and *An. sinensis* is more abundant in the late summer [14]. Therefore, a larger scale information on species composition dynamics over time and distributions would enable in-depth understanding of ecology of Hyrcanus group mosquitoes and malaria epidemiology. With this in mind, the QS protocol developed in this study should facilitate to acquire large scale phenology information, which is fundamental for assessing the impact of climate change on the malaria epidemiology in the Korean Peninsula. Moreover, the principle of QS-based prediction method can be applied to other *Anopheles* spp., e.g., members of the *Anopheles gambiae* complex of sub-Saharan Africa, to estimate the composition ratio of individual species that exhibit different seasonal occurrence, vector competence, and insecticide resistance.

## Conclusions

In this study, a rapid QS-based method for the prediction of species composition ratios in pooled specimens of members of the Hyrcanus Group was developed. Since this protocol can be adapted as a cost-effective high-throughput analysis tool, rapid processing of multiple *Anopheles* spp. samples from multiple geographical areas and time series is feasible for large-scale studies to better understand the ecology, phenology, and epidemiology of *Anopheles* mosquitoes. Together with molecular tools for the detection of *Plasmodium* spp. and insecticide resistance, this two-step prediction protocol will facilitate to elucidate any possible correlations between vector competence and resistance potential of the Hyrcanus Group. In addition, the same principle can be applied for the quantitative analysis of species composition in other morphologically indistinguishable mosquito species complexes or groups, including the *An. gambiae* complex.

## Supplementary Information

**Additional file 1.**GenBank sequences used for phylogenetic tree construction and alignments.

**Additional file 2**. List of amplified standard DNA mixture ratios (%) of *COI *and *ITS2* for regression analysis.

**Additional file 3**. Primer sets used for copy number determination of *Anopheles* Hyrcanus Group.

**Additional file 4**. Phylogenetic tree of *COI* genes from NCBI sequences and collected mosquito specimens. Maximum likelihood tree with log -1673.92 score was obtained from 38 of GenBank sequences.

**Additional file 5**. Phylogenetic tree of *ITS2* genes from NCBI sequences and collected mosquito specimens. Maximum likelihood tree with log -5366.15 score was obtained from 26 GenBank sequences and 6 collected sequences.

**Additional file 6**. Alignment of *COI* of Anopheles Hyrcanus Group. The partial *COI* gene (641 bp) sequences of six Anopheles species were aligned by ClustalW method. Red pins indicate the species-specific nucleotide sequences, and primer sites are marked as red boxes. Species arrangement was based on phylogenetic analysis of *COI* gene in Additional file 3. Color code was given as background for each species. (s = blue, An. sinensis; k = green, An. kleini; b = purple, An. belenrae; l = yellow, An. lesteri; p = red, An. pullus; sd = gray, An. sineroides).

**Additional file 7**. Alignment of *ITS2* of Anopheles Hyrcanus Group. The rDNA *ITS2* region (450 bp) sequences of six Anopheles species were aligned by ClustalW method. Red pins indicate the species-specific nucleotide sequences, and the primer sites are marked as red boxes. Color code was the same as Additional file 5, but the order was rearranged based on the phylogenetic tree in Additional file 4.

**Additional file 8**. Species composition ratios determined by individual genotyping or predicted by QS from the pooled gDNA and pooled mosquito specimens.

## Data Availability

Not applicable.

## Abbreviations

QS: Quantitative sequencing
*COI*: *Cytochrome C oxidase subunit I*
*ITS2*: *Internal transcribed spacer 2*
ROK: Republic of Korea
DMZ: Demilitarized zone
gDNA: Genomic DNA
BLAST: Basic local alignment search tool
ML: Maximum likelihood
NCBI: National Center for Biotechnology Information
