# Supplementary material for: Prediction of species composition ratios in pooled specimens of the Anopheles Hyrcanus group using quantitative sequencing
Source: Malar J. 2021 Aug 6;20:338. doi: 10.1186/s12936-021-03868-y (PMC8349024; doi:10.1186/s12936-021-03868-y)
Supplement: Supplementary file 1 — Additional file 1.GenBank sequences used for phylogenetic tree construction and alignments. [file 12936_2021_3868_MOESM1_ESM.docx]

**Additional file 1.** GenBank sequences used for phylogenetic tree construction and alignments

| COI | | |  | ITS2 | | |
| --- | --- | --- | --- | --- | --- | --- |
| **Species** | **Accession number** | **Length (bp)** |  | **Species** | **Accession number** | **Length (bp)** |
| *An. sinensis* | MG816539.1 | 15410 |  | *An. sinensis* | GU384699.1 | 1648 |
|  | MG816556.1 | 15414 |  |  | GU384700.1 | 1648 |
|  | MH425419.1 | 649 |  |  | GU384698.1 | 1648 |
|  | KX779765.1 | 662 |  |  | GU384693.1 | 1651 |
|  | KX779600.1 | 662 |  |  | GU384697.1 | 1648 |
|  | LC054427.1 | 650 |  | *An. kleini* | GU384716.1 | 1640 |
|  | AB738318.1 | 658 |  |  | GU384715.1 | 1640 |
| *An. kleini* | KT358445.1 | 658 |  |  | GU384714.1 | 1640 |
|  | KX840622.1 | 658 |  |  | GU384713.1 | 1640 |
|  | KX840634.1 | 658 |  | *An. belenrae* | GU384711.1 | 1639 |
|  | KX840635.1 | 658 |  |  | GU384710.1 | 1639 |
|  | KX840633.1 | 658 |  |  | GU384709.1 | 1639 |
|  | GQ265917.1 | 654 |  |  | GU384708.1 | 1639 |
|  | KX840616.1 | 658 |  |  | GU384707.1 | 1639 |
| *An. belenrae* | KT358456.1 | 658 |  | *An. pullus* | GU384706.1 | 1631 |
|  | KT358455.1 | 658 |  |  | GU384705.1 | 1631 |
|  | KX840609.1 | 658 |  |  | GU384704.1 | 1631 |
|  | KF830742.1 | 605 |  |  | GU384703.1 | 1632 |
|  | KF830743.1 | 605 |  |  | GU384702.1 | 1632 |
| *An. pullus* | KX840615.1 | 658 |  | *An. lesteri* | GU384720.1 | 1637 |
|  | KT358449.1 | 658 |  |  | GU384721.1 | 1637 |
|  | KC135892.1 | 658 |  |  | GU384722.1 | 1637 |
|  | KX840614.1 | 658 |  |  | GU384719.1 | 1637 |
|  | KT358450.1 | 658 |  | *An. sineroides* | GU384723.1 | 1624 |
|  | KX840613.1 | 658 |  |  | GU384724.1 | 1624 |
| *An. lesteri* | EU699048.1 | 660 |  |  | GU384725.1 | 1624 |
|  | MK625000 | 658 |  |  |  |  |
|  | AB738151.1 | 658 |  |  |  |  |
|  | KX840611.1 | 658 |  |  |  |  |
|  | EU699046.1 | 660 |  |  |  |  |
|  | KT358452.1 | 658 |  |  |  |  |
|  | KT358453.1 | 658 |  |  |  |  |
| *An. sineroides* | LC054440.1 | 650 |  |  |  |  |
|  | LC054438.1 | 650 |  |  |  |  |
|  | KX840608.1 | 658 |  |  |  |  |
|  | LC054442.1 | 650 |  |  |  |  |
|  | LC054439.1 | 650 |  |  |  |  |
|  | LC054441.1 | 650 |  |  |  |  |
