# Supplementary material for: Prediction of species composition ratios in pooled specimens of the Anopheles Hyrcanus group using quantitative sequencing
Source: Malar J. 2021 Aug 6;20:338. doi: 10.1186/s12936-021-03868-y (PMC8349024; doi:10.1186/s12936-021-03868-y)
Supplement: Supplementary file 2 — Additional file 2. List of amplified standard DNA mixture ratios (%) of COI and ITS2 for regression analysis. [file 12936_2021_3868_MOESM2_ESM.docx]

**Additional file 2.** List of amplified standard DNA mixture ratios (%) of COI and ITS2 for regression analysis

| COI | Species | C-a | C-b | C-c | C-d | C-e | C-f | C-g | C-h | C-i | C-j | C-k | C-l |
| --- | --- | --- | --- | --- | --- | --- | --- | --- | --- | --- | --- | --- | --- |
|  | s+k+b * | 10 | 20 | 30 | 40 | 50 | 60 | 70 |  | 80 |  | 90 |  |
|  | *An. pullus* | 10 | 30 | 20 | 60 | 50 | 40 |  | 70 |  | 80 |  | 90 |
|  | *An. sineroides* | 40 | 50 |  |  |  |  | 30 |  | 20 |  | 10 |  |
|  | others | 40 |  | 50 |  |  |  |  | 30 |  | 20 |  | 10 |
| **ITS2** | **Species** | **I-a** | **I-b** | **I-c** | **I-d** | **I-e** | **I-f** | **I-g** | **I-h** | **I-i** | **I-j** |  |  |
|  | *An. sinensis* | 10 | 20 | 25 | 25 | 33.3 | 50 | 70 | 10 |  | 90 |  |  |
|  | *An. kleini* | 20 | 70 | 25 | 50 | 33.3 | 25 | 10 | 90 | 10 |  |  |  |
|  | *An. belenrae* | 70 | 10 | 50 | 25 | 33.3 | 25 | 20 |  | 90 | 10 |  |  |

* s+k+b: sum of the DNA proportion of *An. sinensis*, *An. kleini* and *An. belenrae*
