# Supplementary material for: Prediction of species composition ratios in pooled specimens of the Anopheles Hyrcanus group using quantitative sequencing
Source: Malar J. 2021 Aug 6;20:338. doi: 10.1186/s12936-021-03868-y (PMC8349024; doi:10.1186/s12936-021-03868-y)
Supplement: Supplementary file 3 — Additional file 3. Primer sets used for copy number determination of Anopheles Hyrcanus Group. [file 12936_2021_3868_MOESM3_ESM.docx]

**Additional file 3.** Primer sets used for copy number validation of *Anopheles* Hyrcanus Group

| Gene | Primer name | Sequence (5’-3’) | Size (bp) |
| --- | --- | --- | --- |
| COI | An_CO1-F | CTTTAAGTATTCTAATTCGAGCTG | 65 |
|  | An-qCOI-R | AATTTGATCATCTCCAATAAAAGC |  |
| ITS2 | skbq28Sr-F | GTCTCACTATCCGTCTTCCTA | 139 |
|  | skbq28Sr-R | ACTATCAAGCAACACGACTCC |  |
| RPS7 | An_qRPS7-F | GTGCGCGAGCTTGAGAAGAA | 115 |
|  | An_qRPS7-R | GTGGTCGCTTCTGCTTGTTG |  |
| RPL8 | An_qRPL8-F | GTCGTATCGACAAGCCCATC | 110 |
|  | An_qRPL8-R | ATGCTCGACGGGGTTCATC |  |
