# Supplementary figures and images for: Prediction of species composition ratios in pooled specimens of the Anopheles Hyrcanus group using quantitative sequencing
Source: Malar J. 2021 Aug 6;20:338. doi: 10.1186/s12936-021-03868-y (PMC8349024; doi:10.1186/s12936-021-03868-y)

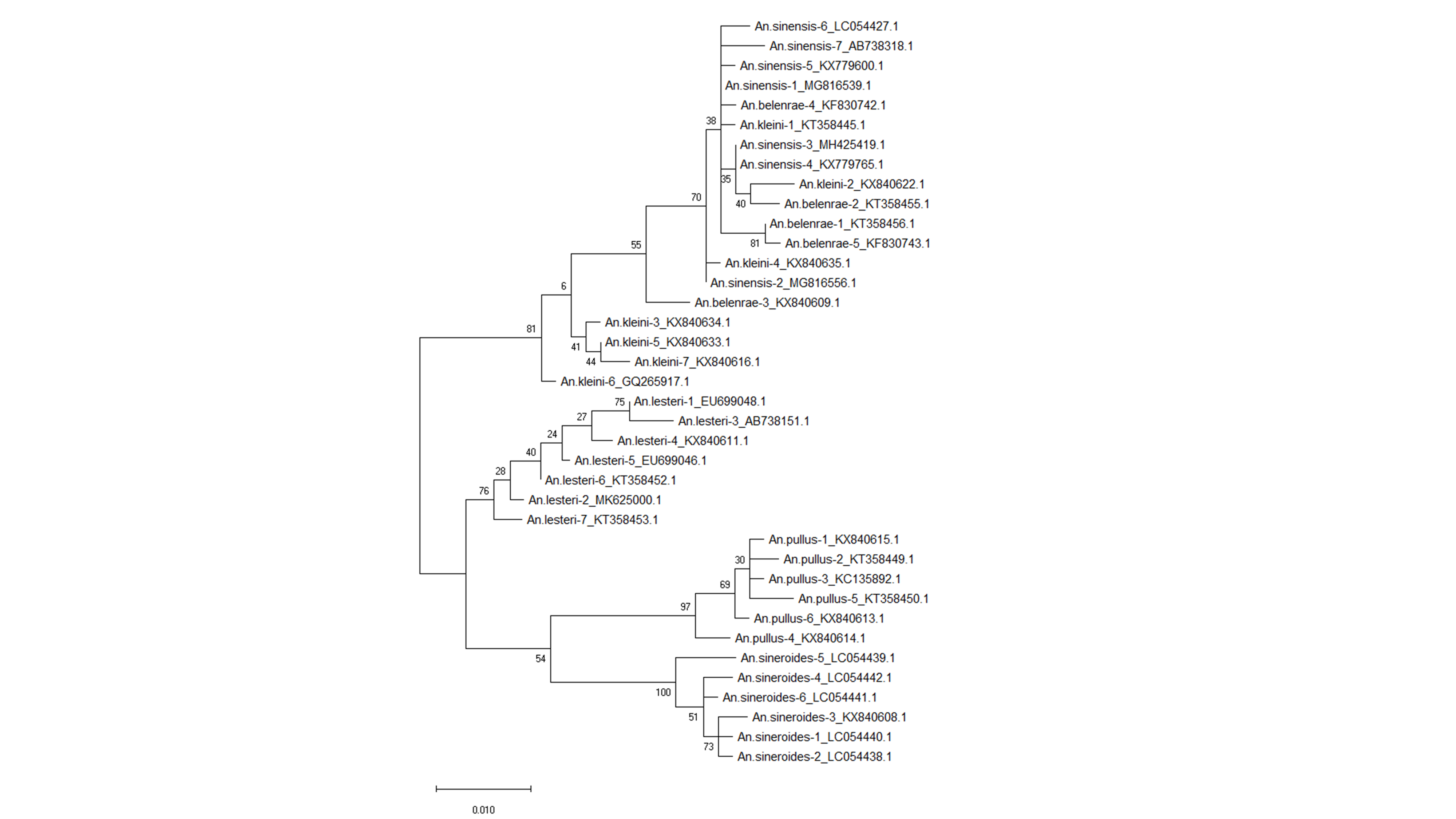

Supplement: Supplementary file 4 — Additional file 4. Phylogenetic tree of COI genes from NCBI sequences and collected mosquito specimens. Maximum likelihood tree with log -1673.92 score was obtained from 38 of GenBank sequences. [file 12936_2021_3868_MOESM4_ESM.tif]

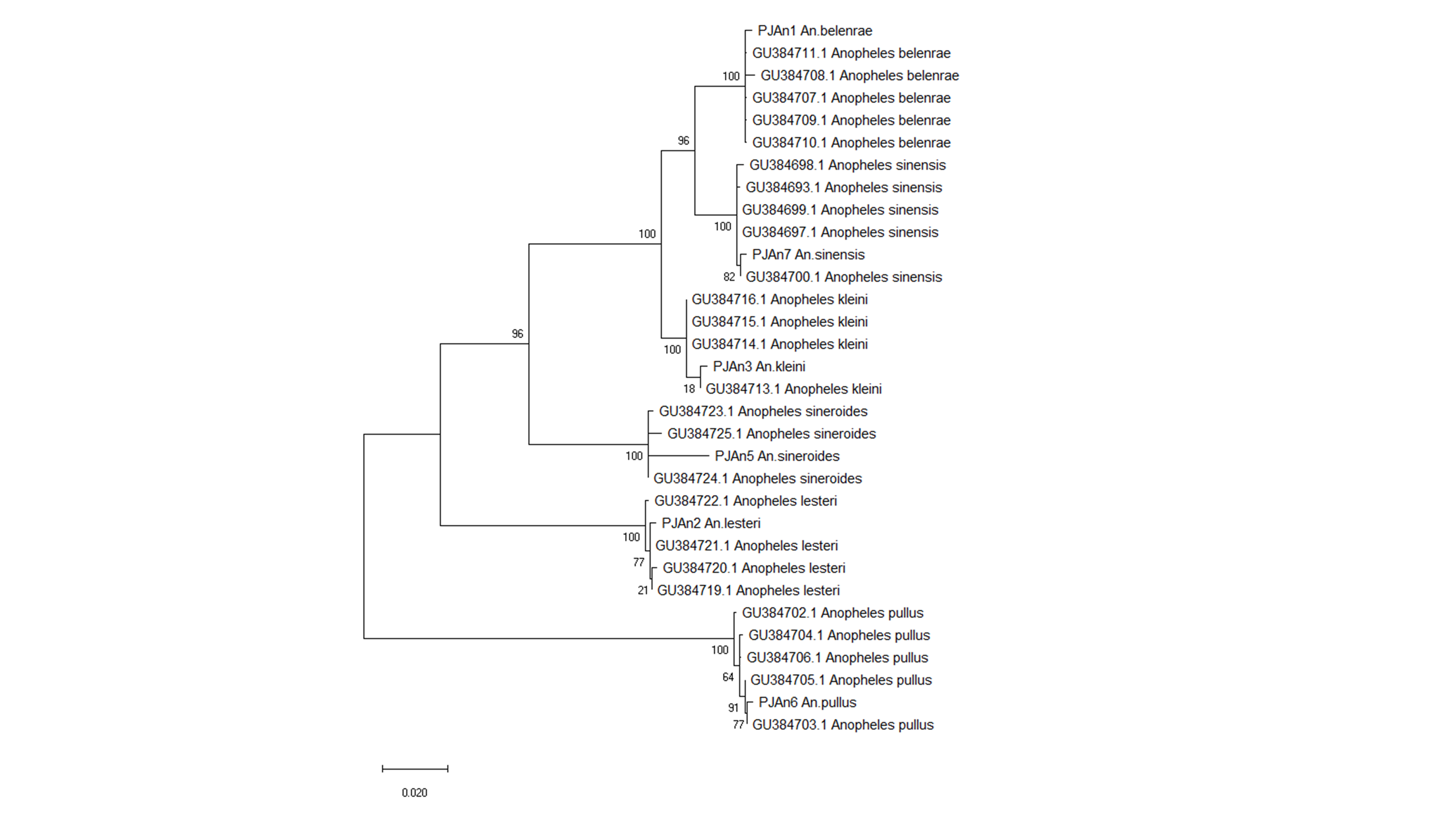

Supplement: Supplementary file 5 — Additional file 5. Phylogenetic tree of ITS2 genes from NCBI sequences and collected mosquito specimens. Maximum likelihood tree with log -5366.15 score was obtained from 26 GenBank sequences and 6 collected sequences. [file 12936_2021_3868_MOESM5_ESM.tif]

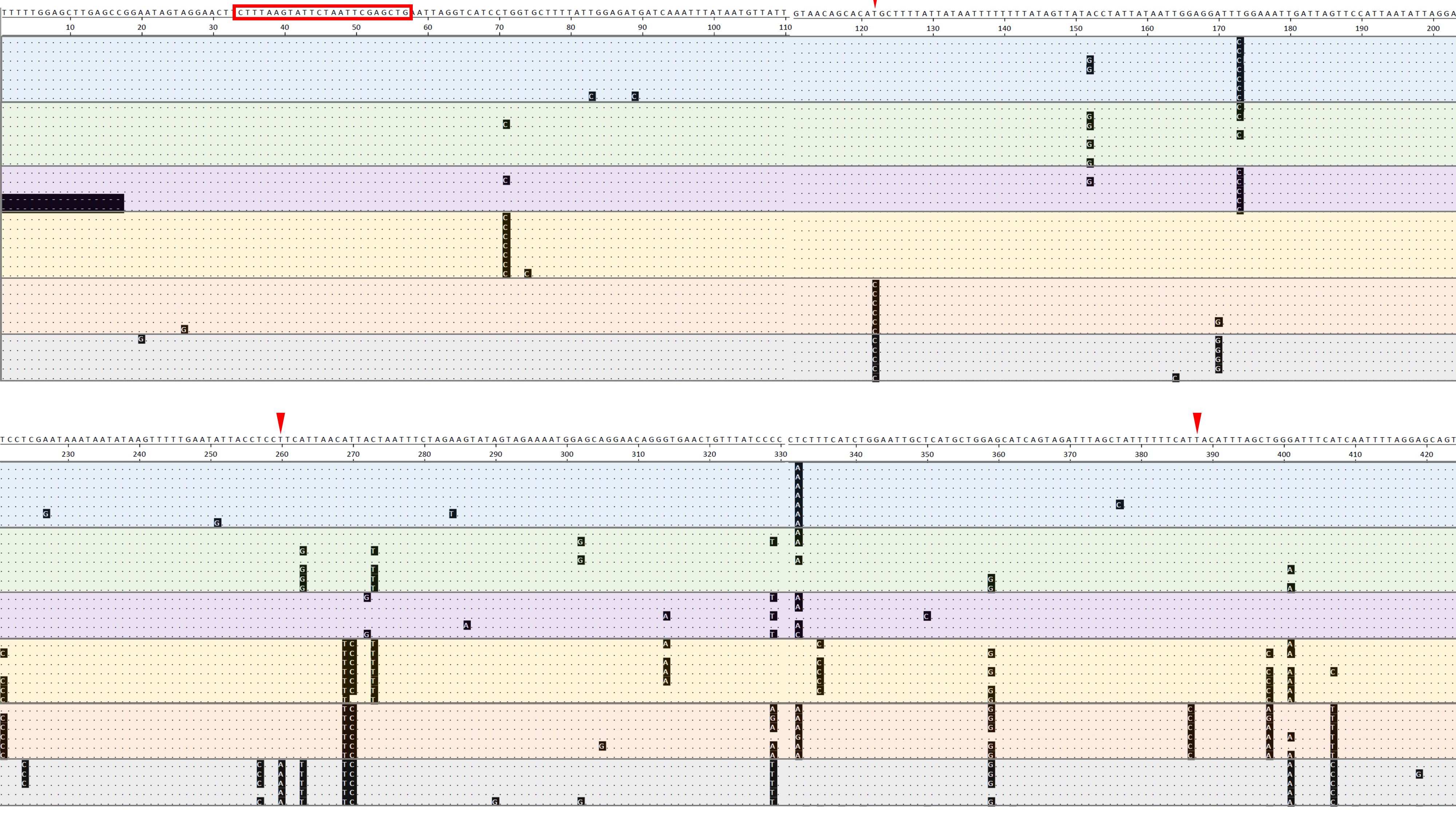

Supplement: Supplementary file 6 — Additional file 6. Alignment of COI of Anopheles Hyrcanus Group. The partial COI gene (641 bp) sequences of six Anopheles species were aligned by ClustalW method. Red pins indicate the species-specific nucleotide sequences, and primer sites are marked as red boxes. Species arrangement was based on phylogenetic analysis of COI gene in Additional file 3. Color code was given as background for each species. (s = blue, An. sinensis; k = green, An. kleini; b = purple, An. belenrae; l = yellow, An. lesteri; p = red, An. pullus; sd = gray, An. sineroides). [file 12936_2021_3868_MOESM6_ESM.tif]

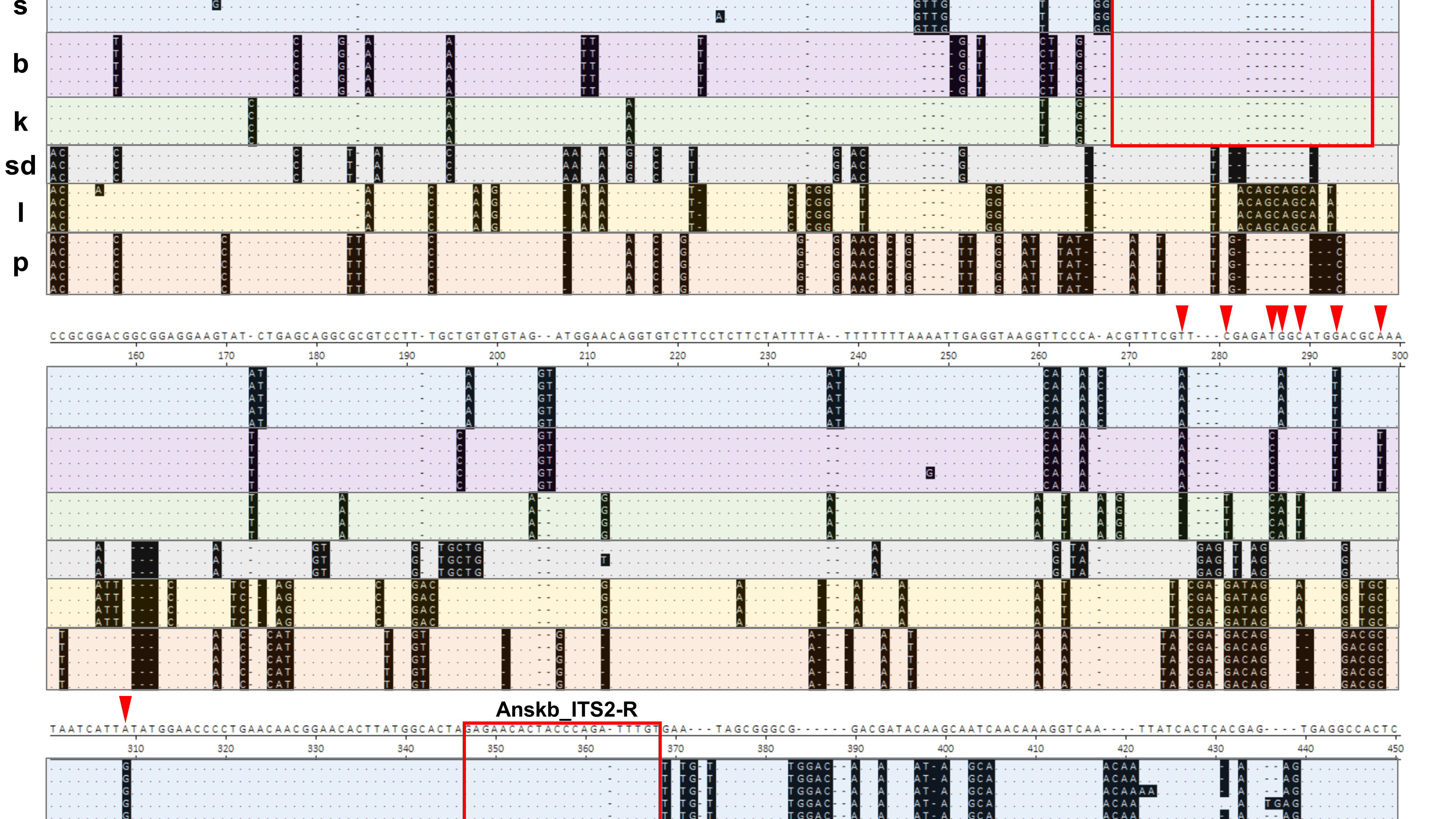

Supplement: Supplementary file 7 — Additional file 7. Alignment of ITS2 of Anopheles Hyrcanus Group. The rDNA ITS2 region (450 bp) sequences of six Anopheles species were aligned by ClustalW method. Red pins indicate the species-specific nucleotide sequences, and the primer sites are marked as red boxes. Color code was the same as Additional file 5, but the order was rearranged based on the phylogenetic tree in Additional file 4. [file 12936_2021_3868_MOESM7_ESM.tif]
