# Supplementary material for: Prediction of species composition ratios in pooled specimens of the Anopheles Hyrcanus group using quantitative sequencing
Source: Malar J. 2021 Aug 6;20:338. doi: 10.1186/s12936-021-03868-y (PMC8349024; doi:10.1186/s12936-021-03868-y)
Supplement: Supplementary file 8 — Additional file 8. Species composition ratios determined by individual genotyping or predicted by QS from the pooled gDNA and pooled mosquito specimens. [file 12936_2021_3868_MOESM8_ESM.docx]

**Additional file 8.** Species composition ratios determined by individual genotyping or predicted by QS from the pooled gDNA and pooled mosquito specimens

| Specimen condiiton | Sample name | Actual value | | | Predicted value | | |
| --- | --- | --- | --- | --- | --- | --- | --- |
|  |  | *An. sinensis*  *(An. belenrae)* | *An. kleini*  *(An. lesteri)* | *An. pullus*  *(An. sineroides)* | *An. sinensis*  *(An. belenrae)* | *An. kleini*  *(An. lesteri)* | *An. pullus*  *(An. sineroides)* |
| Pooled gDNA specimen | 2a | 0.0 | 80.0 | 20.0 | -0.24 | 80.65 | 14.85 |
|  | 2b | 70.0 | 0.0 | 30.0 | 73.11 | -0.40 | 22.93 |
|  | 2c | 0.0 | 60.0 | 40.0 | -0.19 | 63.26 | 36.52 |
|  | 2d | 40.0 | 0.0 | 60.0 | 52.23 | -0.29 | 53.83 |
|  | 2e | 0.0 | 30.0 | 70.0 | -0.12 | 40.76 | 64.34 |
|  | 2f | 20.0 | 0.0 | 80.0 | 27.93 | -0.15 | 75.68 |
|  | 2g | 80.0 | 20.0 | 0.0 | 76.49 | 23.21 | 0.92 |
|  | 2h | 30.0 | 70.0 | 0.0 | 35.66 | 64.03 | 0.92 |
|  | 2i | 60.0 | 40.0 | 0.0 | 60.22 | 39.48 | 0.92 |
|  | 3a | 30.0 | 50.0 | 20.0 | 33.98 | 44.27 | 19.90 |
|  | 3b | 50.0 | 20.0 | 30.0 | 44.85 | 19.69 | 37.34 |
|  | 3c | 20.0 | 30.0 | 50.0 | 25.04 | 28.45 | 47.07 |
|  | 3d | 20.0 | 70.0 | 10.0 | 29.08 | 59.66 | 9.19 |
|  | 3e | 70.0 | 10.0 | 20.0 | 73.73 | 4.96 | 17.62 |
|  | 3f | 10.0 | 20.0 | 70.0 | 21.24 | 19.06 | 64.65 |
| Pooled mosquito specimen | DMZ6 | 0.0 | 66.7 | 12.5 | 0.32 | 53.88 | 15.71 |
|  |  | 16.7 | 0.0 | 4.2 | 17.79 | 2.35 | 4.39 |
|  | DMZ7 | 10.0 | 20.0 | 70.0 | 9.84 | 20.68 | 59.83 |
|  |  | 0.0 | 0.0 | 0.0 | 0.22 | 8.13 | 1.45 |
|  | DMZ8 | 66.7 | 0.0 | 29.2 | 78.81 | -0.44 | 18.27 |
|  |  | 0.0 | 0.0 | 4.2 | 1.56 | -1.85 | 3.63 |
|  | DMZ9 | 10.0 | 0.0 | 90.0 | 10.23 | -0.06 | 89.83 |
|  |  | 0.0 | 0.0 | 0.0 | 0.07 | -1.51 | 1.45 |
|  | PT7 | 37.5 | 0.0 | 62.5 | 41.33 | -0.23 | 63.27 |
|  |  | 0.0 | 0.0 | 0.0 | 0.3 | -6.12 | 1.45 |
|  | PT8 | 60.0 | 40.0 | 0.0 | 57.78 | 42.73 | 0.92 |
|  |  | 0.0 | 0.0 | 0.0 | 0.73 | -3.11 | 1.45 |
